# Phytochemical analysis and radical scavenging profile of juices of *Citrus sinensis, Citrus anrantifolia*, and *Citrus limonum*

**DOI:** 10.1186/2191-2858-4-5

**Published:** 2014-07-07

**Authors:** Abdur Rauf, Ghias Uddin, Jawad Ali

**Affiliations:** 1 Institute of Chemical Sciences, University of Peshawar, Peshawar KPK 25120, Pakistan

**Keywords:** Rutaceae, *Citrus sinensis*, *Citrus anrantifolia*, *Citrus limonum*, Phytochemical screening, Antioxidant activity

## Abstract

**Background:**

The aim of the current investigation was to identify bioactive secondary metabolites including phenols, tannins, flavonoids, terpinedes, and steroids and compare the phytochemical analysis and antioxidant profile of the juice extracted from the fruits of *Citrus sinensis*, *Citrus anrantifolia*, and *Citrus limonum.*

**Results:**

Phytochemical screening is important for the isolation of new, novel, and rare secondary metabolites before bulk extraction. Phytochemical analysis of the desired plant fruits of family Rutaceae revealed the presence of phenols, flavonoids, reducing sugars, steroids, terpinedes and tannins. The fruits of *C. sinensis* and *C. anrantifolia* exhibited the presence of phenols, flavonoids, reducing sugars, steroids, terpinedes and tannins, while the fruits of *C. limonum* indicated the presence of phenols, flavonoids, reducing sugars, terpinedes, and tannins. The fruits of selected plants were also subjected to antioxidant potential by 2,2-diphenyl-1-picrylhydrazyl (DPPH) assay against ascorbic acid at various concentrations. Among the tested plants, *C. sinensis* showed promising antiradical effect (84.81%) which was followed by *C. Anrantifolia* (80.05%) at 100 μg/ml against ascorbic acid (96.36%). The *C. limonum* showed low antioxidant activity among the three selected plants of family Rutaceae.

**Conclusions:**

The current finding is baseline information in the use of the fruits of selected plants as food supplement which may be due to the presence of antioxidant molecules in the family Rutaceae. Further research is needed in this area to isolate the phenolic constituents which possess ideal antiradical potential.

## Background

Recently, there is keen biomedical interest in family Rutaceae (fruits) because their use as raw is mainly associated with low risk of gastric, colorectal, esophageal, and cancer diseases. Citrus is a promising source of vitamin C, folate, and flavonoids due to which citrus is used as a cancer preventing agent [1]. *Citrus sinensis* belong to the family Rutaceae, which is the most widely grown and commercialized species [2]. *C. sinensis* is a rich source of sugars, acids, polysaccharides, and many other phytochemicals such as vitamin C and carotenoids, which provided health benefits against various diseases including cardiovascular and cancer diseases [3,4].

*Citrus anrantifolia* belong to the family Rutaceae and is distributed in tropical and subtropical region. *C. anrantifolia* is commonly used in various traditional systems as an antihelmintic, mosquito repellent, and antiseptic and many other chronic diseases [5]. *Citrus limonum* is also a member of the family Rutaceae. *C. limonum* is a rich source of vitamin C, which is used as folk medicine for the treatment of stomachache, carminative, as antipneumonia, and also for the treatment of dysentery and diarrhea [6]. The current finding deals with the comparative phytochemical analysis for the identification of various classes of secondary metabolites and antioxidant profile of juices of *C. sinensis, C. anrantifolia*, and C*. limonum.*

## Methods

### Plant collection

The fresh fruits of *C. sinensis, C. anrantifolia*, and *C. limonum* were collected from the garden of Institute of Chemical Sciences, University of Peshawar, Peshawar, Pakistan. The fruits of collected plants were stored in the refrigerator of Center of Phytomedicine natural product laboratory. The sample was identified and authenticated by Dr. Abdur Rashid, plants Taxonomist, the voucher no. (PUP714-716) was deposited at the Botany Department, University of Peshawar, Pakistan.

### Place of study

The experimental work was carried out in the Centre for Phytomedicine and Medicinal Organic Chemistry Institute of Chemical Sciences, University of Peshawar, Peshawar, Pakistan.

### Extraction

The fresh Juices of *C. sinensis, C. anrantifolia*, and *C. limonum* were extracted from the fresh fruits and freeze dried and stored in the refrigerator for further analysis.

### Statistical analysis

Data were presented as mean and standard error of means. The statistical analysis was performed using Prism Graphed.

### Chemical and reagents

The ascorbic acid, 2,2-diphenyl-1-picrylhydrazyl (DPPH), and analytical grade methanol were purchased from Merck, Darmstadt, Germany.

### Phytochemical analysis

Chemical test was performed on the juices of *C. sinensis*, *C. anrantifolia*, and *C. limonum* to identify bioactive secondary metabolites according to standard assay procedure.

### DPPH radical scavenging profile

The antioxidant activity of the juices of *C. sinensis*, *C. anrantifolia*, and *C. limonum* was performed by DPPH radical scavenging assay according to standard assay protocol [7]. The positive control used in the current finding was ascorbic acid. The hydrogen atom or electron donation capacities of the juices extracted from fruits and ascorbic acid were measured from the bleaching of the purple-colored methanol solution of DPPH. Experiments were carried out in triplicates. Briefly, a 1-mM solution of DPPH radical solution in methanol was prepared, and 1 ml of this solution was mixed with 3 ml of sample (juices) solutions in methanol (containing 10 to 100 μg) for various fractions (containing 10 to 100 μg) for pure compounds and control (without sample). The solution was allowed to stand for 30 min, in the dark, the absorbance was measured at 517 nm. Decreasing of the DPPH solution absorbance indicates an increase of the DPPH radical scavenging activity. Scavenging of free radicals by DPPH as percent radical scavenging activities (%RSA) was calculated as follows:

$$\begin{array}{l} \%\mathrm{DPPH}=\left(\mathrm{OD}\;\mathrm{control}‒\mathrm{OD}\;\mathrm{sample}\right)\\ \;\times 100/\mathrm{OD}\;\mathrm{control} \end{array}$$

where OD control is the absorbance of the blank sample and OD sample is the absorbance of samples or standard sample.

## Results and discussion

The phytochemical analysis of three selected plants of the family Rutaceae (*C. sinensis, C. anrantifolia*, *C. limonum*) are given in Table 1, while the antiradical effects are listed in Table 2.

**Table 1:** **Antioxidant effect of juices extracted from *C*. *sinensis, C. anrantifolia***, **and *C. limonum***

**Concentration**	**% DPPH *C. sinensis***	**% DPPH *C. anrantifolia***	**% DPPH of *C. limonum***	**Ascorbic acid**
10 μg/ml	49.69 ± 1.11	37.51 ± 1.00	25.47 ± 1.22	91.34 ± 1.22
20 μg/ml	58.46 ± 1.22	43.41 ± 1.90	28.60 ± 1.50	91.39 ± 1.28
30 μg/ml	62.48 ± 1.00	50.94 ± 1.89	34.12 ± 1.29	92.09 ± 1.38
40 μg/ml	65.74 ± 1.98	55.58 ± 1.00	38.51 ± 1.98	92.59 ± 1.99
50 μg/ml	68.13 ± 1.88	61.85 ± 1.24	40.90 ± 1.23	93.35 ± 1.24
60 μg/ml	74.78 ± 1.68	70.38 ± 1.23	50.06 ± 1.86	94.47 ± 1.90
80 μg/ml	81.93 ± 1.22	75.28 ± 1.55	55.45 ± 1.00	95.23 ± 1.98
100 μg/ml	84.81 ± 1.99	80.05 ± 1.90	63.73 ± 1.91	96.36 ± 1.78

**Table 2:** **Phytochemical analysis of juices of *C. sinensis, C. anrantifolia***, **and *C. limonum***

**Chemical constituents**	***C. sinensis***	***C. anrantifolia***	***C. limonum***
Alkaloids	**-**	**-**	**-**
Tannins	**++**	**+++**	**++**
Anthraquinones	**-**	**-**	**-**
Glycosides	**-**	**-**	**-**
Reducing sugar	**+++**	**++**	**+**
Saponins	**-**	**-**	**-**
Flavonoids	**++**	**+**	**+**
Phlobatanins	**-**	**-**	**-**
Steroids	**++**	**+**	**-**
Terpenoids	**+**	**+**	**+**
Phenol	**+++**	**++**	**+**

### Antioxidant effect

The effect of the different plants of the family Rutaceae juices at various concentrations against DPPH is presented in Table 1. All the tested plants (*C. sinensis, C. anrantifolia*, and *C. limonum*) exhibited promising antiradical activity as compared to the standard drug (ascorbic acid). *C. sinensis* showed 84.81% antiradical effect at 100 μg/ml which was followed by *C. anrantifolia* 80.05%, while *C. limonum* 63. 73%. The antioxidant effect of the tested juices was increase in dose-dependent mode.

Phytochemical screening is significant for the isolation of antioxidant natural product from medicinal plants. The fruits of the tested plants of the family Rutaceae exhibited the presence of phenols, flavonoids, reducing sugars, steroids, terpinedes, and tannins. The fruits of *C. sinensis* and *C. anrantifolia* showed the presence of phenols, flavonoids, reducing sugars, steroids, terpinedes, and tannins, while the fruits of *C. limonum* indicated the presence of phenols, flavonoids, reducing sugars, terpinedes, and tannins. Different reactive oxidative species (ROS) including superoxide radicals and hydroxyl (OH) radicals are natural products produced in living organisms [7]. Reactive oxidative species produced as by product play a key role in cell signaling. However, biomolecule oxidation produced excessive ROS which caused major damage to cell structure and resulted to different kinds of diseases such as cancer, stroke, and diabetes. Antioxidants are key inhibitors which produce lipid peroxidation not only for food protection but also as a defense mechanism of living cells against oxidative damage [8,9]. The juices extracted from the fruits of selected plants were also evaluated to antioxidant potential by DPPH assay against ascorbic acid at various concentrations (10 to 100 μg/ml). Among the tested plants, *C. sinensis* showed promising antiradical effect (84.81) which was followed by *C. Anrantifolia* (80.05) at 100 μg/ml against ascorbic acid (96.36). *C. limonum* showed low antioxidant activity among the three selected plants of the family Rutaceae.

## Conclusions

The current finding suggests the use of the fruits of selected plants as food supplement which may be due to the desirable presence of antioxidant molecules in the family Rutaceae. The current finding directed the scientist to isolate new, rare, and novel antioxidant molecules from *C. sinensis*, *C. anrantifolia*, and *C. limonum.*

## Competing interests

The authors declare that they have no competing interests.
